# Molecular Epidemiological Investigation and Genetic Evolution Analysis of Porcine Circovirus 3 in Hunan Province, China, from 2021 to 2024

**DOI:** 10.3390/v18020159

**Published:** 2026-01-24

**Authors:** Yirun Tai, Xiaoming Tang, Jie Fan, Ke Liu, Wenwu Pan, Guoying Sun, Yanli Zhu, Ping Chen, Wenlong Zhao, Zhongxin Fan, Meng Ge

**Affiliations:** 1 College of Veterinary Medicine, Hunan Agricultural University, Changsha 410128, China; 18149072685@163.com (Y.T.); 18574437134@163.com (J.F.); 18876740690@163.com (G.S.); 17394112713@163.com (Y.Z.); cp09151@163.com (P.C.); 15089831730@stu.hunau.edu.cn (W.Z.); 2 Hunan Animal Disease Prevention and Control Center, Changsha 410128, China; vet8111@163.com; 3 Shanghai Veterinary Research Institute, Chinese Academy of Agricultural Science, No. 518, Ziyue Road, Shanghai 200241, China; liuke@shvri.ac.cn; 4 Loudi Animal Disease Prevention and Control Center, Loudi 417000, China; robert1116@163.com

**Keywords:** porcine circovirus 3, molecular epidemiology, genetic evolution, Cap protein, structural prediction

## Abstract

Porcine circovirus 3 (PCV3), first reported in 2016, is associated with diverse clinical conditions, including porcine dermatitis and nephropathy syndrome, reproductive disorders, and systemic inflammation, and affects pigs of all ages. To investigate the prevalence and genetic evolution of PCV3 in Hunan Province, China, 700 lymph node tissue specimens were collected from slaughterhouses and hazard-free disposal centers across 14 prefecture-level cities between 2021 and 2024 and screened using real-time quantitative PCR (qPCR). Epidemiological investigation revealed an overall PCV3 positivity rate of 29.4% (206/700) in the province. The highest prevalence was observed in Yiyang City (56%, 28/50), whereas no positive samples were detected in Zhuzhou City (0/30). Among the positive samples, 34 specimens from different cities with Ct values < 25 were selected for Cap gene amplification and sequencing. Phylogenetic analysis showed that PCV3c was the predominant genotype (67.6%, 23/34), followed by PCV3a (32.4%, 11/34), while PCV3b was not detected. We identified twelve amino acid substitution sites within Cap proteins. Furthermore, B-cell linear epitope prediction and homology modeling of the Cap protein identified seven linear epitopes, with ten amino acid variation sites located within these epitopic regions. This study enriches the molecular epidemiological data of PCV3 in southern China and provides a reference for future PCV3 control strategies.

## 1. Introduction

PCV3, classified within the porcine circovirus family, is characterized as a single-stranded circular DNA virus with a negative-sense genomic organization. Comprising approximately 2000 nucleotides in its compact genome, it ranks among the smallest documented animal viruses [1]. The circular genome architecture of PCV3 features three principal open reading frames: ORF1 and ORF2 are arranged in opposed orientations, flanking the ORF3 region. The Rep protein (Rep) essential for viral replication is encoded by ORF1, while ORF2 directs the synthesis of the structural Cap protein (Cap) essential for virion assembly. Notably, ORF3 encodes a non-structural protein that has been implicated in apoptotic induction capabilities [2,3]. Phylogenetic analyses reveal that PCV3 demonstrates closer evolutionary affinity with chiropteran circoviruses than with other members of the porcine circovirus clade [4].

In 2016, American researcher Palinski performed metagenomic sequencing on specimens from porcine dermatitis and nephropathy syndrome (PDNS)-affected swine and made the first identification of PCV3 [5]. Retrospective analysis by Brazilian investigator Rodrigues demonstrated that PCV3 could be traced to samples dating back to 1966, confirming its prolonged circulation in global swine populations [6]. Current epidemiological surveillance indicates PCV3’s worldwide distribution, with progressively increasing prevalence rates documented across multiple Chinese provinces including Shanxi, Liaoning, Henan, Guangxi, and Jiangsu [7,8,9,10,11,12,13,14]. Pathogenicity studies show that the virus rescued from an infectious PCV3 DNA clone induces PDNS-like pathological changes in piglets during experimental infection [15]. Additionally, histopathological examinations have demonstrated the virus’s broad tissue tropism, with pronounced inflammatory infiltrates particularly evident in pulmonary tissues [16]. The emerging challenge of polymicrobial infections involving PCV3 co-occurrence with pathogens such as Porcine circovirus 2 (PCV2) [14], African swine fever virus (ASFV) [17], and secondary bacterial agents significantly complicates swine health management protocols. These collective findings indicate that PCV3 is a notable factor for herd health, with potential implications for the sustainability of global pork production systems.

In this study, we conducted PCV3 antigen detection and genetic evolution analysis of the viral Cap protein using 700 tissue specimens collected from swine slaughterhouses and Hazard-free disposal centers across 14 prefecture-level cities in Hunan Province, China. This systematic investigation provides baseline data for developing PCV3 prevention strategies and enhances our understanding of the pathogen’s evolutionary characteristics.

## 2. Materials and Methods

### 2.1. Collection

A total of 700 inguinal lymph node(ILNs) tissue specimens were randomly collected from slaughterhouses and hazard-free disposal centers distributed across 14 prefecture-level cities (Loudi, Chenzhou, Changde, Yiyang, Hengyang, Yueyang, Shaoyang, Changsha, Xiangxi, Zhangjiajie, Xiangtan, Huaihua, Yongzhou, and Zhuzhou) in Hunan Province during the period spanning 2021 to 2024. Each sample comes from the lymph node tissue of an individual fattening pig.

### 2.2. Extraction of DNA from the Samples

After supplementing tissue samples with 400 μL of PBS and stainless steel beads (3 mm diameter), mechanical disruption was performed using a homogenizer. Genomic DNA extraction was conducted using a commercial nucleic acid extraction kit (Hangzhou Bori Biotechnology Co., Ltd., Hangzhou, China) following manufacturer’s protocols. We then aliquoted the purified DNA into sterile, nuclease-free 0.2 mL microcentrifuge tubes and cryopreserved them at −80 °C in an ultra-low temperature freezer for long-term storage.

### 2.3. Detection and Analysis of PCV3

Following the protocol established by Franzo et al. [18], all 700 samples were analyzed using qPCR for PCV3 detection. The oligonucleotide sequences were as follows: PCV3-QF (5′-TGACGGAGACGTCGGGAAAT-3′), PCV3-QR (5′-CGGTTTACCCAACCCCATCA-3′), and PCV3-probe (5′-FAM-GGGCGGGGTTTGCGTGATTT-BHQ1-3′), all synthesized by Tsingke (Beijing, China). The 25 μL reaction system consisted of: 12.5 μL PCR master mix, 0.5 μL forward primer, 0.5 μL reverse primer, 0.25 μL TaqMan probe, 5 μL DNA template, supplemented with 6.25 μL sterile nuclease-free water. Geographic visualization of PCV3 distribution in Hunan Province was performed using DataV (https://datav.aliyun.com/portal/school/atlas/area_selector Accessed on 14 April 2025) mapping platform combined with Adobe Illustrator 2023 for graphical refinement. Chi-square (χ^2^) tests were performed using GraphPad Prism (version 9.5) to compare PCV3-positive rates between different sample sources (slaughterhouses versus hazard-free disposal centers). Comparisons among different cities were not subjected to χ^2^ testing due to uneven and, in some cases, limited sample sizes; instead, regional differences were evaluated descriptively based on observed positivity rates. A two-sided *p* value < 0.05 was considered statistically significant for the χ^2^ analysis.

### 2.4. Amplification, Sequencing, and Analysis of the Cap Site

Based on the PCV3 whole-genome sequences available in GenBank (Table 1), we designed a pair of species-specific primers (PCV3-Cap-F: 5′-GCGTTGGGGGGGGGGTATTTA-3′; PCV3-Cap-R: 5′-GCCCGGCACCAAAATGAGACACAG-3′) targeting conserved regions flanking mutation sites of the Cap gene, which were synthesized by Tsingke (Beijing, China). We performed amplicon sequencing of the Cap gene on PCV3-positive samples (with Ct values < 25) that were randomly selected from each city, ensuring geographical representation. The sequencing was conducted by Sangong (Shanghai, China). Sequence analysis confirmed the amplified Cap gene fragment length as 645 nucleotides.

The acquired Amino acid sequence underwent alignment with reference sequences (Table 1) using MEGA11 (version 11.0.8) software suite. Multiple sequence alignments were performed via ClustalW algorithm, followed by phylogenetic reconstruction through maximum likelihood method with 1000 bootstrap replicates in MEGA11. Linear B-cell epitopes within Cap proteins were predicted using BepiPred-3.0 prediction server (https://services.healthtech.dtu.dk/services/BepiPred-3.0/ Access on 20 February 2025).

## 3. Results

### 3.1. Prevalence of PCV3 in Hunan Province

A total of 700 ILNs tissue samples collected from slaughterhouses and hazard-free disposal centers across 14 prefecture-level cities in Hunan Province between 2021 and 2024 were screened for PCV3. The results revealed an overall provincial positivity rate of 29.4% (206/700), with notable regional variations: Yiyang City exhibited the highest prevalence at 56%, while no positive cases were detected in Zhuzhou City (Table 2). Chi-square analysis performed using GraphPad Prism revealed that the PCV3-positive rate in samples from hazard-free disposal centers was significantly higher than that in slaughterhouses (46.3%, 88/190 vs. 23.1%, 118/510; χ^2^ = 35.81, *p* < 0.0001).

**Figure 1:**
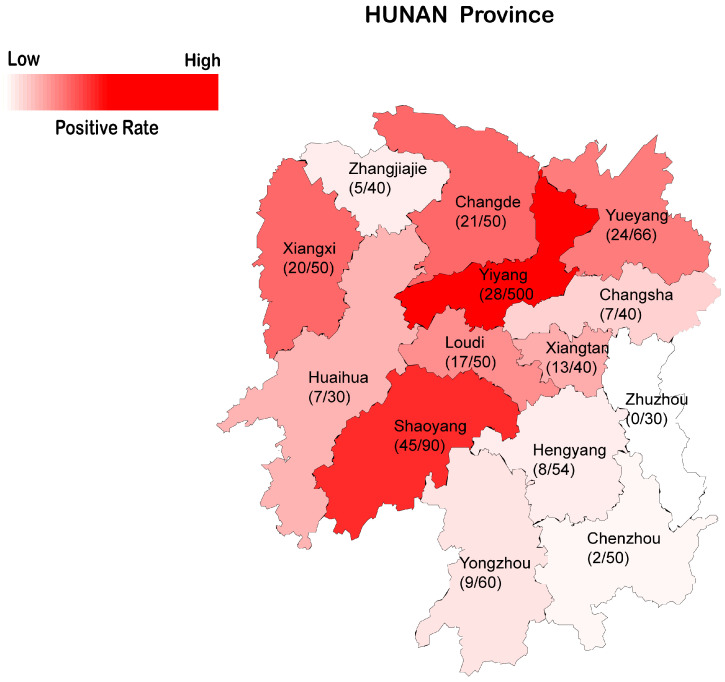
A distribution map of PCV3-positive rate in Hunan Province of China, with decreasing positive rates represented by a gradient from deep red to light red.

Geospatial analysis further demonstrated distinct epidemiological patterns. As illustrated in Figure 1, PCV3 positivity rates displayed a pronounced geographic gradient, characterized by elevated rates in northern and western regions compared to lower rates in southern and eastern areas. These interregional disparities may correlate with variations in swine production systems, socioeconomic development levels, Geographical topographic differences and regional biosecurity protocols.

### 3.2. Genetic Evolution Analysis of PCV3 Cap Site

**Figure 2:**
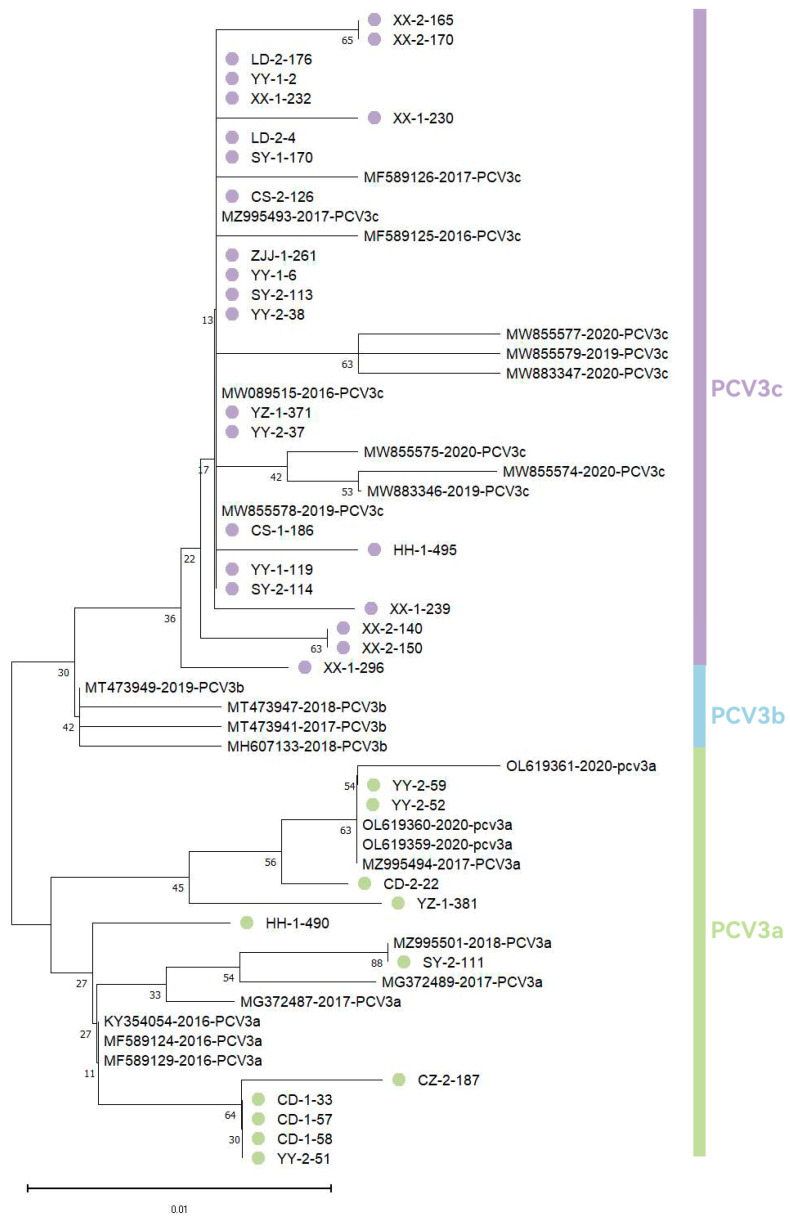
Phylogenetic analysis of PCV3 Cap protein sequences was performed using the maximum likelihood method in MEGA 11, based on 34 representative PCV3-positive samples selected from Hunan Province. The sequences obtained in this study are represented by circles, with purple circles indicating PCV3c and green circles indicating PCV3a. The newly generated Cap gene sequences have been submitted to GenBank, and accession numbers will be provided upon assignment.

We conducted genetic evolution analysis of the PCV3 Cap protein sequences by constructing a phylogenetic tree that included 34 sequenced samples randomly selected from qPCR-positive cases from various cities in Hunan Province. (Xiangxi: *n* = 8, Yiyang: *n* = 7, Shaoyang: *n* = 4, Changde: *n* = 4, Yueyang: *n* = 1, Loudi: *n* = 2, Huaihua: *n* = 2, Changsha: *n* = 2, Yongzhou: *n* = 2, Zhangjiajie: *n* = 1, Chenzhou: *n* = 1), along with reference Cap protein sequences of different viral lineages obtained from NCBI. Phylogenetic analysis (Figure 2) revealed two distinct PCV3 genotypes circulating in Hunan Province: PCV3a (32.4%, 11/34) and PCV3c (67.6%, 23/34). The predominant genotype was PCV3c, representing the major epidemic strain in this region, while PCV3a maintained a minor presence. Notably, PCV3b was absent from our sampled population.

**Figure 3:**
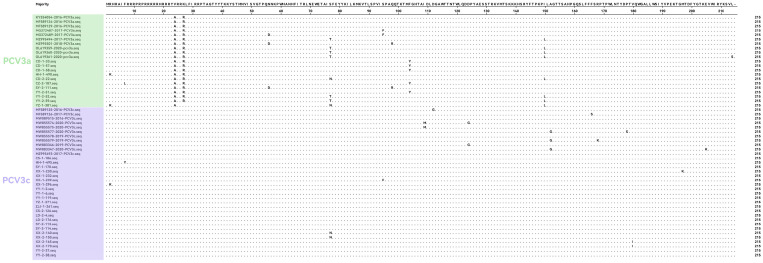
Alignment analysis of 34 Cap protein sequences was performed using MegAlign  software that available in DNASTAR 11.1, with PCV3a and PCV3c genotypes, respectively, shown in green and purple.

To investigate the genetic variability of PCV3 Cap protein in Hunan Province, we performed comparative analysis between 34 newly sequenced isolates and historical reference sequences from NCBI. As demonstrated in Figure 3, twelve amino acid variant sites were identified in the Cap protein region, most of the difference sites were located at the N-terminal of the PCV3 Cap protein, while the C-terminal was relatively conserved. Notably, three non-synonymous mutations (S77N/T, F104Y, and I150L) showed distinct evolutionary patterns: variations at positions 77 and 150 exhibited temporal progression across multiple sampling periods, while the 104 substitution was uniquely detected in current sequencing data.

### 3.3. Molecular Characterization Prediction of PCV3 Cap Protein

**Figure 4:**
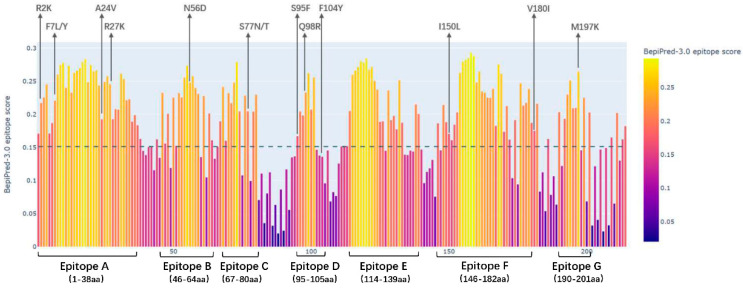
B–cell linear epitopes prediction for the PCV3 Cap protein was performed using BepiPred-3.0 (threshold = 0.15), with scores decreasing along the gradient from yellow to purple. Regions scoring above 0.15 were identified as linear epitopes. A total of seven B–cell linear epitopes (epitopes A–G) were predicted, with amino acid mutation sites on these epitopes marked by arrows above the plot.

The BepiPred-3.0 web server (https://services.healthtech.dtu.dk/services/BepiPred-3.0/ Access on 20 February 2025) was employed to predict B-cell linear epitopes in the PCV3 Cap protein from sequenced samples. As presented in Figure 4, seven linear epitopes were identified using the BepiPred-3.0 algorithm, including Epitope A (1–38 aa), Epitope B (46–64 aa), Epitope C (67–80 aa), Epitope D (95–105 aa), Epitope E (114–139 aa), Epitope F (146–182 aa), and Epitope G (190–201 aa).

### 3.4. Prediction and Analysis of PCV3 Cap Protein Structure

**Figure 5:**
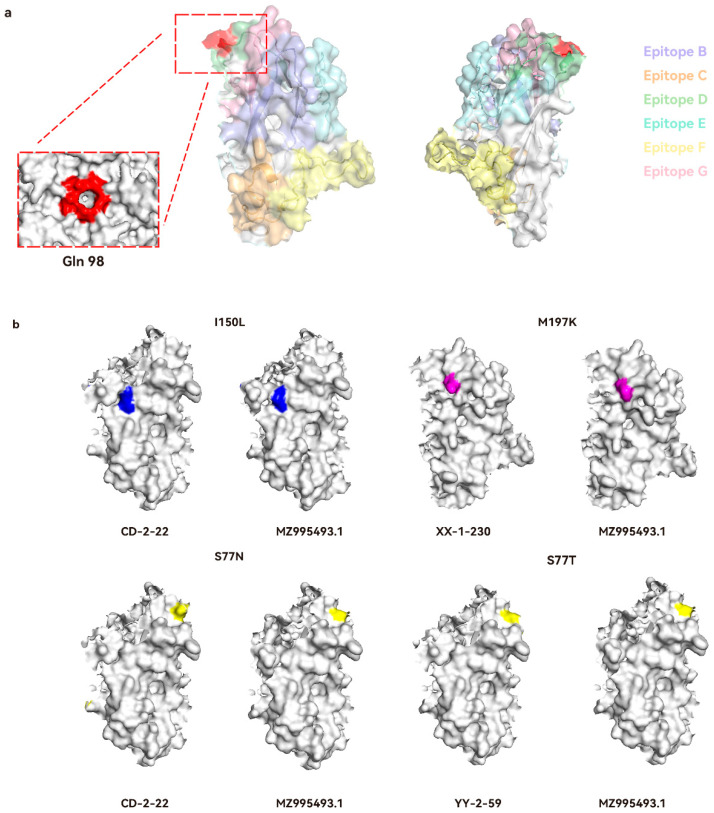
Structural prediction of the PCV3 Cap protein. (**a**) This prediction of the PCV3 Cap protein monomeric structure, with the red-boxed site being amino acid position 98 on the multimeric structure, (**b**) Homology modeling comparison before and after mutation at amino acid divergent positions 77, 150, and 197 in this study. Structural prediction was performed using SWISS-MODEL, with visualization conducted in PyMOL.

The structural prediction of PCV3 Cap protein was performed using SWISS-MODEL (Access on 20 February 2025) and PyMOL (version 3.0), with B-cell epitopes and amino acid variant sites subsequently annotated on the predicted structure (Figure 5a). Eight amino acid mutation sites were identified on the Cap protein surface. Notably, residue 98 was found localized at the multimeric interface of the Cap protein structure, spanning a substantial surface area. Furthermore, structural analysis revealed that mutation at position 77 to asparagine (N) exhibited a more pronounced protrusion compared to the threonine (T) variant (Figure 5b).

## 4. Discussion

In recent years, PCV3 has been widely distributed across China, with certain genetic diversity observed among the strains circulating in different regions. Meanwhile, subclinical infections have been identified in pig herds [19], posing a potential threat to the swine industry. Therefore, a clear understanding of PCV3’s prevalence in Hunan Province is valuable for disease surveillance and scientific disease control in the region.

In this study, we investigated the prevalence and genetic evolution of PCV3 in Hunan Province, analyzing 700 lymph node tissue samples collected from slaughterhouses and Hazard-free disposal center across 14 prefecture-level cities using qPCR. The findings revealed an overall PCV3 detection rate of 29.4% (206/700) in the province, with individual city-level prevalence ranging from 0% to 56%. Notably, some samples exhibited low Ct values (<17) in PCV3-positive samples, indicating high viral loads. Furthermore, the detection rate in hazard-free disposal centers (46.3%, 88/190) was higher than that in slaughterhouses (23.1%, 118/510). This distinct prevalence profile could be influenced by sampling bias, as disposal centers inherently receive pigs with overt health issues. Beyond this potential bias, we hypothesize that PCV3 may operate through two plausible pathogenic mechanisms: it could act as a primary pathogen by inducing host immunosuppression and facilitating secondary infections through impaired immune surveillance; alternatively, it might proliferate as an opportunistic pathogen, exploiting states of immune compromise or environmental stressors triggered by primary infections to exacerbate disease severity. The real-time PCR assay applied in this study has been routinely used and previously validated in our laboratory for PCV3 detection [20]. It should be noted that the real-time PCR assay employed in this study was primarily used as a screening tool for PCV3 detection. Given the genetic diversity of PCV3, primer- or probe-dependent variability may theoretically influence detection sensitivity for certain viral variants. However, this potential limitation is unlikely to affect the overall conclusions of this study, as subsequent phylogenetic and evolutionary analyses were conducted exclusively using high-quality ORF2 sequence data obtained from samples with low Ct values.

Geospatial analysis revealed distinct epidemiological patterns: the northwestern and central regions exhibited higher infection rates, potentially associated with intensive swine farming practices and high-density livestock transportation networks. Conversely, lower prevalence observed in eastern areas may be associated with urbanization characteristics and enhanced biosecurity protocols. Given the limited sample size from Zhuzhou City, the absence of PCV3-positive samples should be interpreted cautiously and does not necessarily indicate the absence of viral circulation. These findings suggest spatial heterogeneity in PCV3 epidemiology that may be influenced by regional and management-related factors, with potential implications for swine health.

Several studies indicate that amino acid mutations at positions 24 (A24) and 27 (R27) within the nuclear localization signal (NLS) region, a motif critical for viral nuclear entry, contribute to host immune evasion, establishing these positions as crucial molecular markers for PCV3 genotyping [21]. Current classification divides PCV3 into three genotypes: PCV3a (A24R27), PCV3b (A24K27), and PCV3c (V24K27). Epidemiological surveillance data reveal distinct geographical distributions of these genotypes in China. Fu et al. [21] reported PCV3 detection rates of 21.5%, 27.8%, and 31.1% in southern provinces (Guangxi, Guangdong, Jiangxi, Fujian, and Hunan) during 2015–2017, with genotype prevalence being PCV3c (48%) > PCV3a (42%) > PCV3b (10%). Subsequent national surveillance by Ge et al. [22] detected PCV3 in 42.87% of 2568 porcine samples from 17 provinces during 2019–2020, with PCV3c predominating (86.96%). More recent data from Wang M-H et al. [16] demonstrated variable infection rates (21.4–37.9%) across six breeding facilities in five regions from 2020 to 2021, revealing distinct genotype distributions: PCV3b in Heilongjiang and Hebei, PCV3a in Xinjiang, and PCV3c in Hubei and Henan.

Our findings in Hunan Province align with previous reports by Ku et al. [23], showed PCV3c as the predominant strain (followed by PCV3a), with no PCV3b detected. The absence of PCV3b in this study may reflect regional evolutionary characteristics of PCV3, as suggested by the heterogeneous genotype distributions reported across different provinces. However, this observation may also be influenced by the inherent limitations of our sampling framework, as samples were collected exclusively from slaughterhouses or hazard-free disposal centers within a single province. A more comprehensive understanding of PCV3 regional evolution would require large-scale, nationwide epidemiological investigations.

With respect to the predominance of PCV3c, current genotyping schemes are largely based on amino acid substitutions at positions 24 and 27 of the Cap protein. Therefore, the dominance of PCV3c observed in this study may be associated with mutations at these residues, although this interpretation remains speculative and requires experimental validation. Currently, it remains unknown whether there is cross-protection between different PCV3 genotypes and how co-infections with other pathogens affect the viral replication and mutation Capabilities. Therefore, control strategies for PCV3 require further research. Notably, we observed a phylogenetic discrepancy in the YZ-1-381 sample: despite displaying A24 and K27 amino acids characteristic of PCV3b, this strain clustered with PCV3a in the Cap gene phylogeny. The typing methodology for PCV3 needs further investigation and standardization to enable more accurate analysis of its genetic evolution.

To better understand PCV3, we investigated its Cap protein, informed by prior research on PCV2 counterpart. Previous studies on PCV2 have demonstrated that single amino acid substitutions at positions 131 and 190 within the defined linear epitopes of the Cap protein can alter viral neutralization phenotypes. Notably, simultaneous mutations at residues 131 and 191 produce an additive effect on neutralization efficacy [24]. This understanding of PCV2 Cap protein modulation of viral antigenicity and pathogenesis lays the groundwork for exploring parallel mechanisms in PCV3 [25,26]. Through BepiPred-3.0 analysis, we identified seven putative B-cell linear epitopes on the PCV3 Cap protein. This prediction aligns with the epitopes 57NKPWH61 and 161QSLFFF166 previously characterized by Jiang et al. [27] using dot blot and peptide ELISA techniques. Our sequencing analysis of PCV3 strains revealed twelve amino acid variation sites, with ten located within predicted linear epitope regions. Structural analysis showed that variations at positions 56, 77, 98, and 197 (surface-exposed protrusions) and positions 95, 104, 150, and 180 (concave surface regions) exhibit distinct spatial distributions. Of particular interest is residue 98, which is located at the multimeric interface of the Cap protein. Spatial conformational changes at this site might interfere with neutralizing antibody recognition, and alterations in charge polarity could potentially impact the stability of the viral capsid multimer. These structure-function relationships require further experimental validation. Notably, several mutations identified in this study (e.g., S77N/T, F104Y, and I150L) were located within predicted epitope regions, suggesting that they may potentially influence Cap protein antigenicity or antibody recognition. However, these inferences are based on sequence and structural predictions, and their effects on neutralizing antibody binding or immune evasion remain to be experimentally confirmed. Evolutionary analysis indicated positive selection at residues 24 and 77, suggesting a potential important role in viral adaptation. The location of these positively selected sites in immunodominant regions implies that they may contribute to viral adaptability to host immune defenses, rendering them notable targets for future vaccine and diagnostic design [15,28,29].

## 5. Conclusions

In conclusion, this study systematically monitored and analyzed the epidemiological profile of PCV3 in selected slaughterhouses and Hazard-free disposal centers across Hunan Province during 2021–2024. The findings contribute to the molecular epidemiological database of PCV3 in this region and provide valuable observations on its genetic evolution patterns. These surveillance data establish a useful foundation, primarily for ongoing disease surveillance, and offer a reference for future research on PCV3 control.

## Figures and Tables

**Table 1:** Reference strain information of PCV3.

Accession No	Origin	Genotypes	Year
MF589129.1	CN-Hunan	PCV3a	2016
KY354054.1	CN-Hunan	PCV3a	2016
MF589124.1	CN-Hunan	PCV3a	2016
MG372487.1	CN-Hunan	PCV3a	2017
MG372489.1	CN-Hunan	PCV3a	2017
MZ995494.1	CN-Hunan	PCV3a	2017
MZ995501.1	CN-Hunan	PCV3a	2018
OL619359.1	CN-Hunan	PCV3a	2020
OL619360.1	CN-Hunan	PCV3a	2020
OL619361.1	CN-Hunan	PCV3a	2020
MF589125.1	CN-Hunan	PCV3c	2016
MW089515.1	CN-Hunan	PCV3c	2016
MF589126.1	CN-Hunan	PCV3c	2017
MZ995493.1	CN-Hunan	PCV3c	2017
MW855578.1	CN-Hunan	PCV3c	2019
MW855579.1	CN-Hunan	PCV3c	2019
MW883346.1	CN-Hunan	PCV3c	2019
MW855574.1	CN-Hunan	PCV3c	2020
MW855575.1	CN-Hunan	PCV3c	2020
MW855577.1	CN-Hunan	PCV3c	2020
MW883347.1	CN-Hunan	PCV3c	2020
MH607133.1	CH	PCV3b	2018
MT473941.1	CN-SD	PCV3b	2017
MT473947.1	CN-SD	PCV3b	2018
MT473949.1	CN-SD	PCV3b	2019

**Table 2:** PCV3 Detection Results in Prefecture-level Cities Across Hunan Province.

	Sample Type	Samples	PCV3 Positive Samples	PCV3 Positive Rate	Total Samples	Total Positive Samples	Total Positive Rate
Yiyang	Slaughterhouse	30	13	43.3%	50	28	56.0%
	Hazard-free disposal centers	20	15	75%			
Shaoyang	Slaughterhouse	60	20	33.3%	90	45	50.0%
	Hazard-free disposal centers	30	25	83.3%			
Changde	Slaughterhouse	30	12	40%	50	21	42.0%
	Hazard-free disposal centers	20	9	45%			
Xiangxi	Slaughterhouse	30	4	13.3%	50	20	40.0%
	Hazard-free disposal centers	20	16	80%			
Yueyang	Slaughterhouse	56	17	30.4%	66	24	36.4%
	Hazard-free disposal centers	10	7	70%			
Loudi	Slaughterhouse	30	11	36.7%	50	17	34.0%
	Hazard-free disposal centers	20	6	30%			
Xiangtan	Slaughterhouse	30	8	26.7%	40	13	32.5%
	Hazard-free disposal centers	10	5	50%			
Huaihua	Slaughterhouse	30	7	23.3%	30	7	23.3%
Changsha	Slaughterhouse	30	4	13.3%	40	7	17.5%
	Hazard-free disposal centers	10	3	30%			
Yongzhou	Slaughterhouse	60	9	15%	60	9	15.0%
Hengyang	Slaughterhouse	34	8	23.5%	54	8	14.8%
	Hazard-free disposal centers	20	0	0%			
Zhangjiajie	Slaughterhouse	30	5	16.7%	40	5	12.5%
	Hazard-free disposal centers	10	0	0%			
Chenzhou	Slaughterhouse	30	0	0%	50	2	4.0%
	Hazard-free disposal centers	20	2	10%			
Zhuzhou	Slaughterhouse	30	0	0%	30	0	0.0%
Total	Slaughterhouse	510	118	23.1%	700	206	29.4%
	Hazard-free disposal centers	190	88	46.3%			

## Data Availability

The nucleotide sequences generated in this study have been submitted to the GenBank database. Accession numbers will be provided upon assignment. Other data supporting the findings of this study are available from the corresponding authors upon reasonable request.
